# Eye tracking technology in endoscopy: Looking to the future

**DOI:** 10.1111/den.14461

**Published:** 2022-11-27

**Authors:** Arun Sivananthan, Jabed Ahmed, Alexandros Kogkas, George Mylonas, Ara Darzi, Nisha Patel

**Affiliations:** ^1^ Institute of Global Health Innovation Imperial College London London UK; ^2^ The Hamlyn Centre Imperial College London London UK; ^3^ Imperial College NHS Healthcare Trust London UK

**Keywords:** adenoma detection, colorectal cancer, endoscopic technology, gaze analysis

## Abstract

The visual patterns of an endoscopist, that is, what the endoscopist is looking at during luminal endoscopy, is an interesting area with an evolving evidence base. The tools required for gaze analysis have become cheaper and more easily accessible. A comprehensive literature search was undertaken identifying 19 relevant papers. Gaze analysis has been used to identify certain visual patterns associated with higher polyp detection rates. There have also been increasing applications of gaze analysis as an objective study tool to compare the effectiveness of endoscopic imaging technologies. Gaze analysis also has the potential to be incorporated into endoscopic training. Eye movements have been used to control and steer a robotic endoscope. This review presents the current evidence available in this novel and evolving field of endoscopic research.

## INTRODUCTION

Endoscopy is the gold standard investigation to identify and resect adenomatous polyps.^1^ An increase in adenoma detection rate (ADR) by 1% has been shown to reduce colorectal cancer risk by 3%.^2^

There are multiple factors contributing to successful adenoma detection. Technological improvements such as high‐definition colonoscopy and imaging technologies including computer aided diagnostic endoscopy (CADe) improve the detection of lesions^3^ and endoscopic adjuncts such as EndoCuff improve the exposure of lesions with a demonstrable improvement of ADR.^4^, ^5^

The visual patterns of an endoscopist, that is, what the endoscopist is looking at during luminal examination of the colon, is an interesting area of endoscopy with an evolving evidence base. Endoscopists' visual gaze patterns are paramount in the detection of colonic pathology. The eye movements of an endoscopist during colonoscopy can be assessed by gaze analysis. Research using gaze analysis is allowing us a greater insight into how visual patterns differ between experts with higher ADR and nonexperts with lower detection rates.

**Figure 1:**
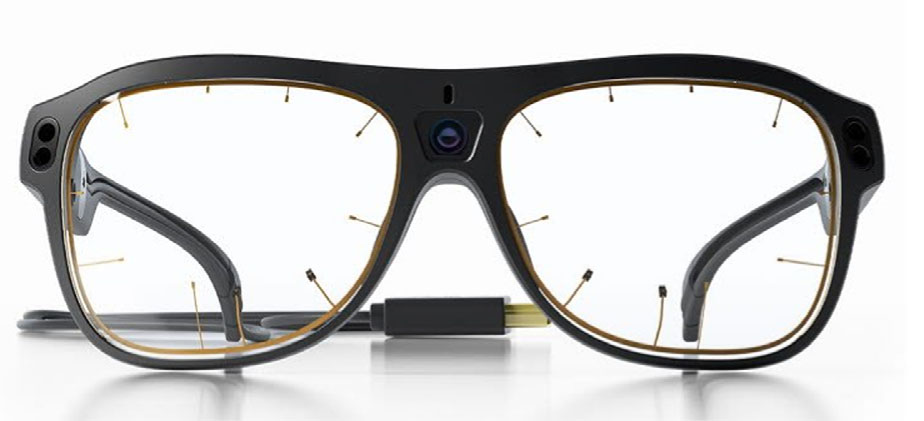
Eye tracking glasses. Tobii pro glasses 3 (Tobii, Stockholm, Sweden).^7^

The tools required for gaze analysis have become cheaper and more easily accessible over the last decade or so. Invasive techniques from the 1960s such as contact lenses equipped with mirrors^6^ have been superseded by simpler systems using eye tracking glasses (Fig. 1) or standing cameras.

Gaze analysis has also been utilized successfully in other areas of medicine. In neuropsychiatry, gaze analysis during tasks, such as saccades, smooth pursuit, and visual search, can help diagnose conditions such as schizophrenia,^8^ Parkinson's disease, and attention deficit hyperactivity disorder.^9^

**Figure 2:**
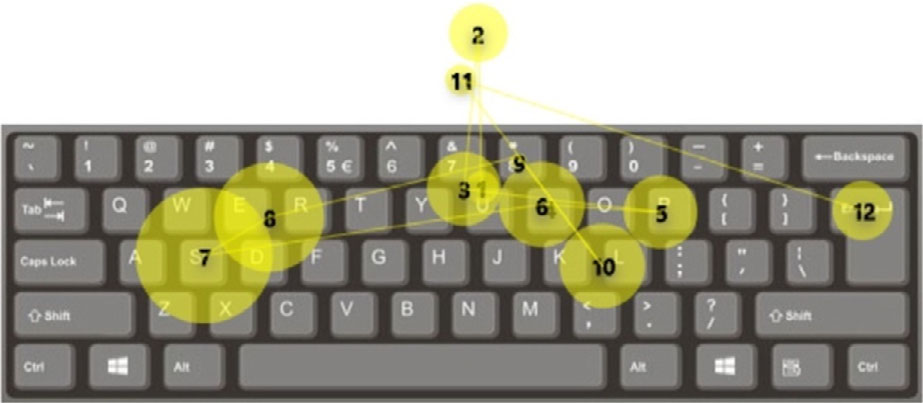
Example of a scan‐path during use of a gaze‐contingent keyboard.^11^ (Reproduced from Harezlak and Kasprowski^11^ with permission.)

Eye movements can also be used therapeutically. Eye tracking glasses can be employed to utilize eye movements as a control input, that is, looking right would be an equivalent input as a joystick being pushed right, known as “gaze control.” The application of gaze‐control technology described above has been used to improve the quality of life in those who cannot communicate due to conditions such as “locked‐in syndrome.” Interfaces using gaze control allow the selection of letters on a screen^10^ (Fig. 2). After training, users can reach 20 words per minute, resulting in books being written by gaze control alone.

This review reports the research available on gaze analysis and gaze control in endoscopy.

## STUDY OVERVIEW

The preferred reporting items for systematic reviews and meta‐analyses (PRISMA) methodology was employed. Consent or ethical/institutional review were not required as this was a literature review. The literature search aims to evaluate the current evidence available in the use of eye gaze and gaze analysis/eye tracking and its potential to improve gastrointestinal endoscopy.

## RESEARCH METHODS

The PubMed, Cochrane Library, and Embase databases were searched until July 20, 2022 to identify relevant research articles. The following keywords were used: Gaze (OR Gaze Analysis OR Eye Tracking OR Gaze Tracking OR Visual Patterns OR Gaze Control) AND Endoscopy (OR Esophagogastroduodenoscopy OR Oesophagogastroduodenoscopy OR Colonoscopy OR Sigmoidoscopy OR Capsule Endoscopy) (Appendix S1).

Two authors participated in the literature search and study selection (AS and JA). Disagreements were discussed with another author (NP). Studies on human subjects that were relevant to the question, peer reviewed, and in the English language were included. Studies in a language other than English, not relevant, or duplicate publications were excluded.

## RESULTS

**Figure 3:**
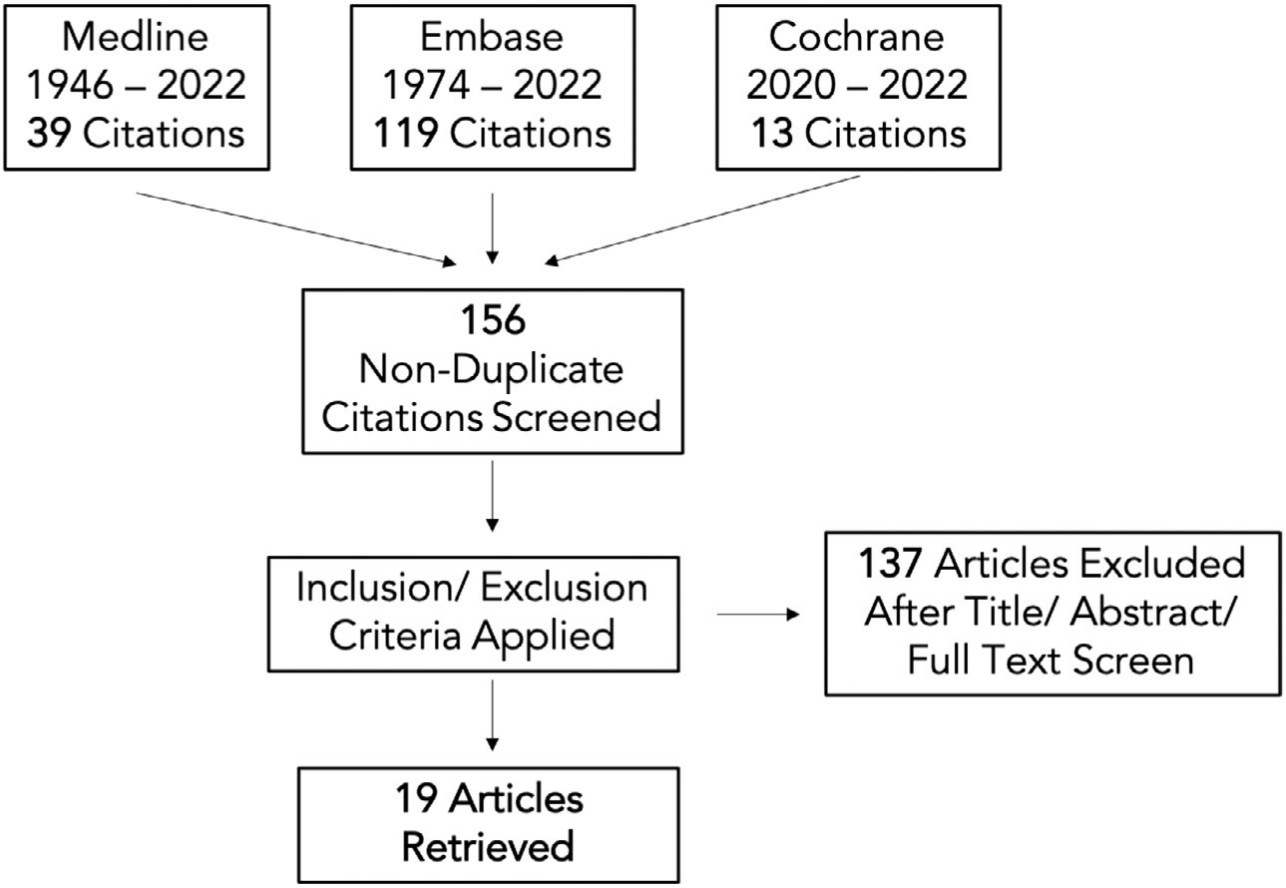
Study selection flowchart: 19 articles were retrieved after literature review search.

We identified 19 eligible studies. The studies were grouped into: “visual search strategies for polyp detection”, “keeping a luminal view”, “endoscopy training”, “objective assessment of lesion detection”, and “gaze control”. The study selection flowchart is shown in Figure 3.

### Visual search strategies for polyp detection

Successful identification of polyps is multifactorial with visual search strategies representing one of these factors. Gaze analysis has suggested that increased gaze in the peripheries, of both the endoscopy monitor and bowel lumen, is associated with a higher lesion detection rate and in more experienced endoscopists.^12^, ^13^

Lami *et al*.^12^ examined the visual search strategies of 18 endoscopists and correlated this to their polyp detection rate (PDR). Eye tracking glasses were worn to record gaze during 2 min of withdrawal from the cecum. Fixations (maintenance of gaze on a single location) were recorded (number and duration) in each area of interest. Data were recorded both anatomically (a three by three grid with the lumen corresponding to the center of the grid) and screen based (a three by three grid with the center of the screen corresponding to the center of the grid). The data were analyzed against the endoscopists' PDR.

There was a positive correlation with gaze in the outer ring and higher PDR. A greater number of fixations and longer durations in the outer ring was associated with a higher PDR. This was for both the anatomical (fixation count: *r* = 0.56, *P* = 0.02; fixation time: *r* = 0.62, *P* < 0.01) and screen based analysis (fixation count: *r* = 0.55, *P* = 0.02; fixation time: *r* = 0.56, *P* = 0.02). There was similarly a significant correlation between PDR and time spent focusing on the monitor vs. off the monitor.^12^

**Figure 4:**
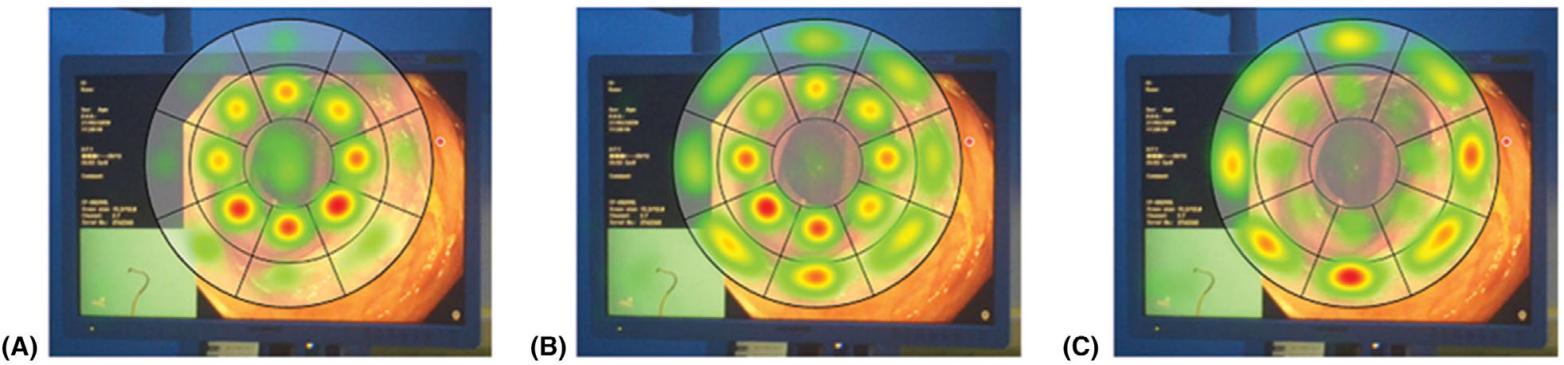
Positive correlation between fixation frequency in peripheries vs. total procedure weighted score.^13^ Heat map shows cumulative fixation distribution: (A) the lowest scoring eight colonoscopies, (B) the middle scoring 10 colonoscopies, and (C) the highest scoring eight colonoscopies (from bottom left to top right of the graph). (Reproduced from Karamchandani *et al.* ^13^ with permission.)

A study from the same group analyzed the gaze patterns in 12 trainee endoscopists completing 24 colonoscopies in total.^13^ The endoscopies were objectively scored by an independent assessor using a directly observed procedural score devised by the Joint Advisory Group on gastrointestinal endoscopy (scored between 1 and 20). This showed a positive correlation between fixation frequency on the outer circle (of both lumen and screen) with a higher procedure score (Fig. 4).

**Figure 5:**
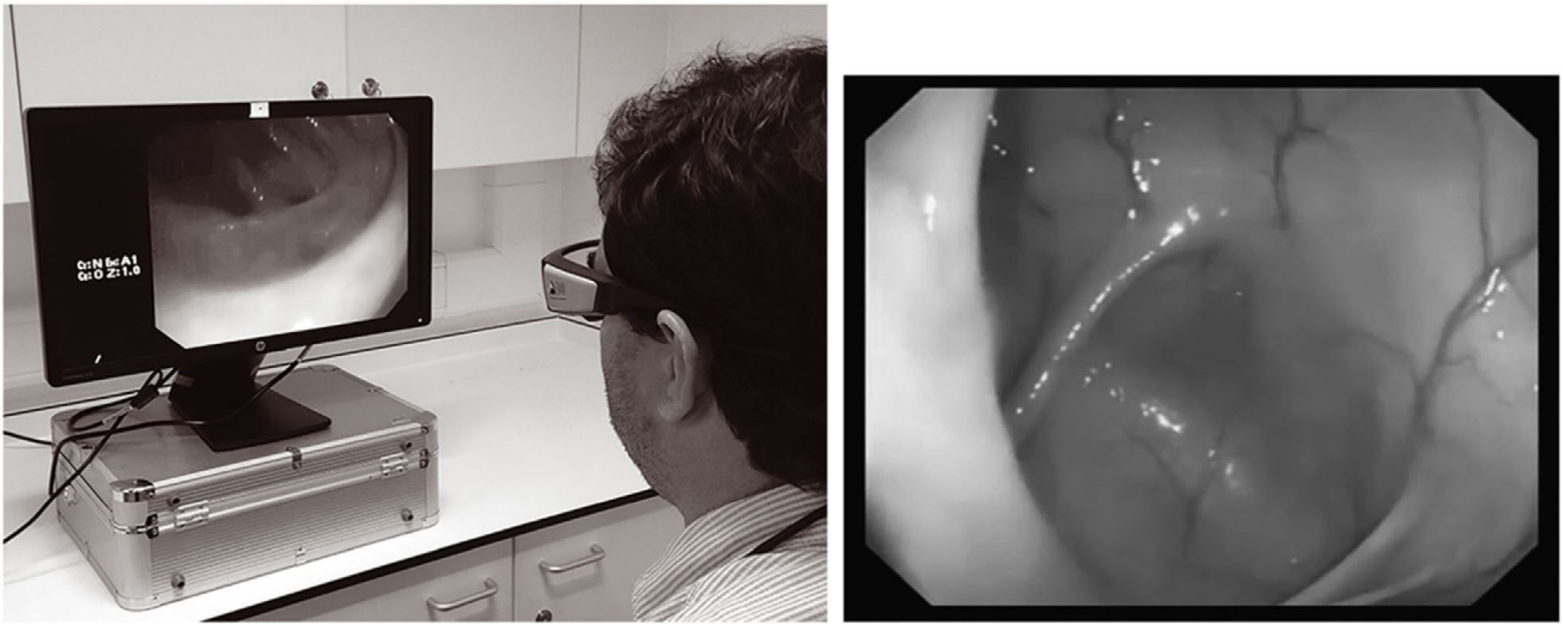
Withdrawal video observation. Left, user wearing eye tracking glasses observing withdrawal video; right, hepatic flexure on left side of screen.^14^

This was further supported by a trial comparing the gaze patterns of 20 novice and 14 experienced endoscopists during observation of a video of withdrawal through the hepatic flexure (Fig. 5).^14^

The study reported that experienced endoscopists had a significantly higher percentage of peripheral fixations (13.4% vs. 23.0%, *P* = 0.013). Of interest, they also found experienced endoscopists had a significantly greater percentage of left sided fixations during withdrawal through the hepatic flexure (18.6% vs. 33.5%, *P* = 0.005) in keeping with more active search of the poorly seen left bend of the hepatic flexure.^15^

Of note, an older study from 2010 provided conflicting results with 11 endoscopists watching 3 min videos of a colonoscopy withdrawal. ADR was significantly correlated with increased percentage and mean gaze time in the central segment of the screen (*r* = 0.67, *P* = 0.024 and *r* = 0.70, *P* = 0.017, respectively). Interestingly there was a negative correlation between years of experience and the percentage of central gaze time (*r* = −0.67, *P* = 0.025).^16^ These conflicting results could be put down to the small sample size and earlier use of gaze analysis.

Patel *et al*.^17^ in a recent 2022 prospective study of 33 endoscopists showed that as PDR increased there was a trend for lower total duration of fixations on the central lumen.

Wenzke *et al*. demonstrated the evolution of visual gaze patterns with time by comparing nine first year endoscopy fellows with themselves 2 years later. The mean gaze time and number of fixations were 2–2.53 × higher in the periphery than the center.^18^

Early work comparing three experts and novices watching colonoscopy videos suggested eye movement speed was faster in experts (33.3 pixels/frame ± 2.7) than in trainees (26.7 ± 1.0) (*P* = 0.03), although this was a relatively early and small trial.^19^

Visual gaze patterns seen in upper gastrointestinal (UGI) endoscopy have also been studied. A pilot study looked at five endoscopists examining a total of 35 patients UGI tracts. Three endoscopists had completed more than 1000 esophagogastroduodenoscopies (EGDs) and two had completed under 1000 EGDs. Heat maps were generated of the endoscopists to represented areas of greatest fixation and there appeared to be a suggestion that the more experienced endoscopists spent greater time in “blind spots” such as the “greater curvature of the upper part, posterior wall of the body, and lesser curvature of the antrum”. However, with such small numbers, no statistically significant evaluation was achieved, but it does nonetheless show the feasibility of visual gaze analysis to help identify the visual gaze patterns of expert endoscopists vs. novices in UGI endoscopy.^20^

These trials suggest that expert endoscopists spend more of their visual search in the peripheries of the screen. They also show that in more poorly visualized areas, such as the hepatic flexure or the posterior body of the stomach, there is a corresponding adjustment in visual search strategy by experts that is not seen by novices. It is important to note, however, these are low power studies with heterogenous methodologies but with nonetheless compelling signals to more effective visual search strategies.

### Keeping a luminal view

Maintaining luminal colonic views is essential to allow complete and thorough examination of the mucosa and thus successful lesion detection. The colon is tortuous, and peristalsis and mucosal folds make maintenance of a clear luminal view challenging.

A study using gaze analysis and simulated colonoscopy aimed to identify the differing gaze patterns, between expert and novice endoscopists, during loss of a luminal view.^21^

**Figure 6:**
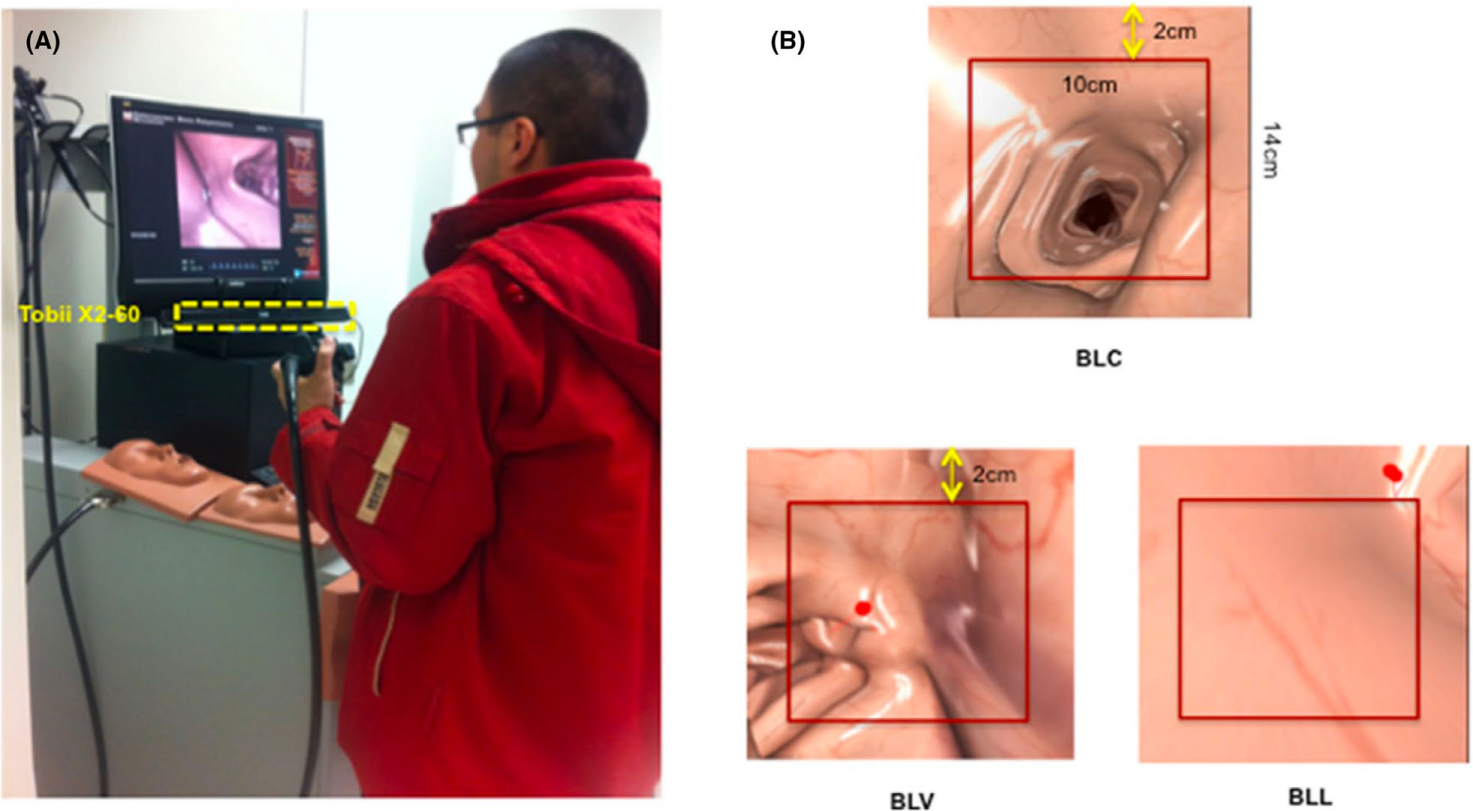
Bowel lumen views. (A) Experimental setup using simulator and fixed camera. (B) Bowel lumen in the center of the screen (BLC), bowel lumen at the edge of the screen but visible (BLV), or bowel lumen lost from the screen (BLL).^21^ (Reproduced from He *et al*.^21^ with permission.)

Twenty‐six endoscopists (six expert and 20 novice) performed simulated virtual reality endoscopies whilst wearing eye tracking glasses. The image on the screen was defined either as bowel lumen in the center of the screen, bowel lumen at the edge of the screen but visible, or bowel lumen lost from the screen entirely (Fig. 6).^21^

The time in each category (as a percentage of total time) and the number of transitions between the differing screen views were recorded. In addition, the saccadic rate was noted.

Expert endoscopists spent a significantly higher proportion of the endoscopy with the lumen centered (bowel lumen in the center of the screen = 70% vs. 51%, *P* = 0.108). When the lumen was at the side of the screen experts were significantly more likely to recenter the lumen than novices (*n* = 7, 29% vs. *n* = 125, 18%, *P* < 0.001). Concurrently, novices were more likely to move from the lumen at the side of the screen to loss of the luminal view (*n* = 61, 9% vs. *n* = 0, 0%).

When the bowel lumen was at the edge of the screen expert endoscopists demonstrated a significantly higher saccadic rate than when the lumen was centered than when the luminal view was lost (5.81 ± 1.57 count/s vs. 4.73 ± 1.31 count/s) but there was no significant difference in novices (4.08 ± 1.49 count/s; 4.20 ± 1.41 count/s).

In summary, expert endoscopists appeared more able to transition from losing the luminal view back to the luminal view using a more active visual search. The capability of transitioning from the bowel lumen at the edge of the screen to the center of the screen may be a useful objective marker of endoscopic expertise.

### Endoscopy training

Endoscopy training is time consuming and involves hours of coaching, instructing, and mentoring. In the context of the recent pandemic there is a significant backlog of endoscopy with endoscopy training falling behind.^22^

Gaze analysis has been shown to be a potentially valuable training adjunct which, in combination with a deep learning algorithm, may be able to replicate some aspects of training without a trainer being present.^23^

Eight surgical trainees in early colonoscopy training (<10 procedures) were asked to perform a colonoscopy on a virtual reality simulator wearing eye tracking glasses. The colonoscopy monitor view was recorded and the navigation losses (loss of luminal view) were labeled.

At the point of navigation loss (51 in total), gaze patterns were analyzed. Fixations and saccades were recorded during navigation loss and reorientation. Using these data points and a deep learning algorithm a system was developed for alerting the user at the point of navigation loss with accuracy (91.80%), sensitivity (90.91%), and specificity (94.12%).

As a result, the group are creating an automated training algorithm that can deliver appropriate instruction during navigation loss.^23^ Such a system would be beneficial to trainees who could receive valuable training without reliance on a trainer. Systems such as this may also be useful for ongoing training or maintenance of certified endoscopists after completion of endoscopy training.

### Objective assessment of lesion detection

Endoscopy is a rapidly evolving field with regular introduction of new imaging technologies many of which are focused on improved lesion detection as an outcome.

Evaluation of such technologies have been assessed by subjective reporting of lesion detection. Gaze analysis offers an accurate objective assessment of such outcomes. When lesions are detected the eye movements towards the lesion confirm objectively both lesion detection and the time to see the lesion.

**Figure 7:**
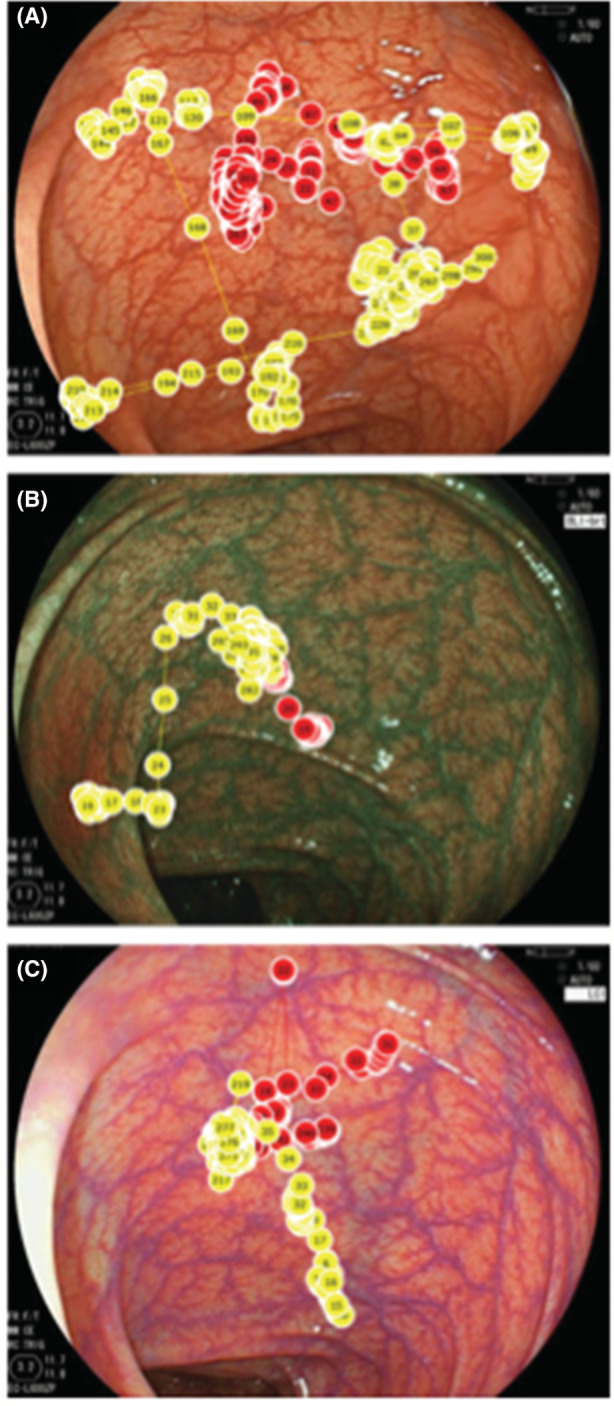
Gaze plot of expert (red spots) and nonexpert (yellow spots). (A) Using white‐light imaging. (B) Blue laser imaging. (C) Linked‐color imaging.^24^

This approach was used to objectively compare three different imaging technologies.^24^ Ten endoscopists reviewed 30 images with white‐light imaging (WLI), blue laser imaging (BLI) (Fujifilm Co., Tokyo, Japan), and link‐colored imaging (LCI) (Fujifilm Co.) from the same luminal viewpoint (Fig. 7).^24^ A fixed eye tracker was attached to the laptop to capture eye gaze data. Detection time was defined as when the participant's eyes stopped at the lesion: 12.6% of lesions were missed with WLI, 6.0% with BLI‐bright, and 4.3% with LCI bright. LCI and BLI‐bright miss rates were significantly lower than that of WLI (*P* < 0.01). Mean detection times were 1.58 (±1.60 s) for WLI, 1.01 (±1.21 s) for BLI‐bright, and 1.10 (±1.16 s) for LCI. Detection time for BLI‐bright and LCI was significantly shorter than that for WLI (*P* < 0.0001).^24^

A similar approach was used to compare the effectiveness of dual red imaging (DRI) vs. WLI to identify source of bleeding during endoscopic submucosal dissection (ESD). Four experienced endoscopists who had completed at least 100 ESDs were randomly shown 40 video cases of bleeding, 20 with DRI and 20 with WLI, whilst wearing eye tracking glasses. The distance of eye movement every 20 ms was calculated giving a measure of eye movement distance per time.

Mean eye movement distance was significantly lower in the DRI group vs. the WLI group for each endoscopist (3.93 ± 0.44 vs. 5.43 ± 0.41 pixels [*P* = 0.0116], 2.07 ± 0.24 vs. 2.92 ± 0.22 pixels [*P* = 0.0081], 1.53 ± 0.23 vs. 2.21 ± 0.21 pixels [*P* = 0.0381], and 1.47 ± 0.23 vs. 2.02 ± 0.19 pixels [*P* = 0.0174], respectively). This suggested that DRI allowed easier identification of the source of bleeding during ESD than WLI.^25^

There have also been three trials to assess CADe systems with visual gaze analysis as an objective assessment tool.

Ten endoscopists watched 50 video clips. Clips were viewed 1 week apart in a random order with and without a CADe system whilst wearing eye tracking glasses. The “time to first fixation” (TTFF), i.e., the time it took for the endoscopist to fixate on a lesion after it appeared on screen, was recorded. The mean TTFF was significantly shorter with CADe vs. WLI (0.690.49 s vs. 0.850.62 s; *P* < 0.01).^26^

A simulated perfect CADe system was simulated using a video with artificial green boxes placed around polyps when detected. Two collages of colonoscopy clips were created with and without the “CADe” system. Nine fellows were randomly assigned to watch both videos 1 week apart whilst wearing eye tracking glasses. Polyp visualization rate (PVR) and time to visualize (TTV) were calculated using gaze analysis. PVR and TTV were both significantly improved and quicker when using the ‘CADe’ system (93.1% ± 0.14% vs. 81.14% ± 0.23%, 0.95 s ± 0.0088 s vs. 1.53 s ± 0.021 s).^27^

Gaze analysis was used to compare the impact of a commercially available CADe system (GI Genius, version March 2020; Medtronic GmbH, Meerbusch, Germany) on visual gaze patterns when false positives from real recordings of CADe use were included. Seventeen participants observed 29 videos both with and without CADe boxes shown, with a 3‐week washout period in between, whilst wearing eye tracking glasses. Seventeen videos contained a single polyp with 12 showing false positive detections. Novices and experts were compared.

All participants' eyes traveled significantly shorter distances with the CADe system (232.68 cm [99% confidence interval (CI) 210.43–262.33]) vs. without (248.86 cm [99% CI 221.39–282.88]) but of interest this was similarly significant in the videos with false positive alerts (329.76 cm [99% CI 293.81–387.00] vs. 368.95 cm [99% CI 331.75–395.10]).^28^

These studies demonstrate the research value of gaze analysis as an objective study tool to use in comparing the multitude of endoscopy technologies that purport to improve lesion detection but rely on subjective reporting to assess this. Objective evaluation, using gaze analysis, can help determine the true value of novel polyp detection technologies such as image enhancement, artificial intelligence, and endoscope adjuncts.

### Gaze control – eye movements as an endoscope steering input

The remit of endoscopy continues to expand to more complex therapeutics such as third space endoscopy including ESD. These methods require fine precision control and significant training due to the procedural complexity and high risk of complications. Several robotic control systems have been developed to allow precision control.^29^

**Figure 8:**
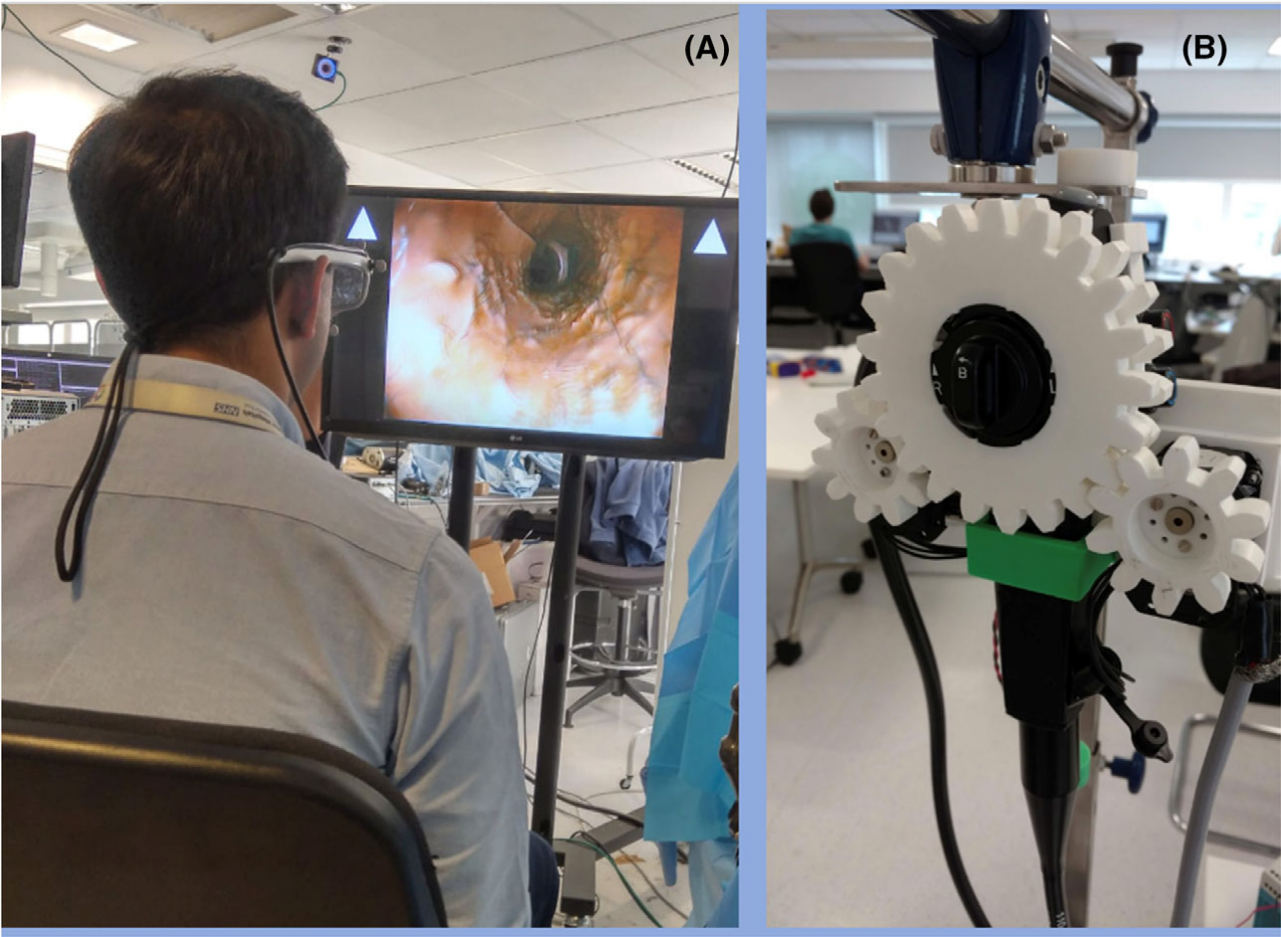
Setup of gaze control system. (A) User wearing eye tracking glasses facing monitor. (B) Motorized 3D printed wheels overlying an endoscope to translate eye movements into endoscope movements.

Our group have conducted a benchtop feasibility trial of a robotic gaze controlled system.^30^ The operator wears eye tracking glasses, which in combination with custom software, custom gears placed over the endoscopy steering wheels, and a robotic arm allows endoscope control by eye movements alone^31^, ^32^ (Fig. 8). This provides the potential for hands‐free endoscopic navigation freeing up both hands for therapeutic instrumentation, which has advantages over the multiple user robotic systems being released currently.

## CONCLUSION

Improved adenoma detection is an important target in the reduction of colorectal cancer. Endoscopy is rapidly evolving through novel technologies to improve detection and exposure of lesions. The application of gaze analysis to colonoscopy is a relatively new field of research.

Early trials have shown interesting signals to higher yield search strategies such as focusing on the peripheries of the lumen and left side of the hepatic flexure during withdrawal, although these trials are small and heterogenous in methodology. Insights from gaze analysis of experienced endoscopists vs. trainees may offer us useful targets for endoscopy training. Analysis of eye movements have suggested objectively assessable differences between expert and novice endoscopists when attempting to keep a luminal view during colonoscopy.

Gaze analysis can also be used to accurately record the exact time to detect a lesion, which is a more valuable objective measure than relying on subjective endoscopist feedback when assessing new endoscopy technologies aimed at improved adenoma or bleeding vessel detection.

Eye tracking glasses can be used as a novel steering system for endoscopes, allowing the endoscopist bimanual freedom for instrumentation.

Gaze analysis and control applied to endoscopy has exciting potential to contribute to the evolution of endoscopy and its critical role in preventing the rising global burden of gastrointestinal cancer. Gaze analysis is a recent and novel area of research and as such studies currently available are small and inconclusive.

## CONFLICT OF INTEREST

Authors declare no conflict of interest for this article.

## FUNDING INFORMATION

None.

## Supporting information


**Appendix S1** Eye gaze literature review: search strategy.
