# Supplementary material for: Eye tracking technology in endoscopy: Looking to the future
Source: Dig Endosc. 2022 Nov 27;35(3):314–22. doi: 10.1111/den.14461 (PMC12136275; doi:10.1111/den.14461)

**Supplementary File 1 - Eye Gaze Literature Review – Search Strategy**

1. Aims:

The literature search aims to evaluate the current evidence available in the use of eye gaze and gaze analysis/ eye tracking and its potential to improve gastrointestinal endoscopy.

1. Search questions:

***Supplementary Table 1: Search Question PICO***

| Question 1 | | MeSH terms |
| --- | --- | --- |
| **Population** | GI endoscopy (simulated or human) |  |
| **Intervention** | gaze analysis/ eye tracking | Gaze, Gaze Analysis, Eye Tracking, Gaze Tracking, Visual Patterns, Gaze Control |
| **Comparator group** | standard endoscopy |  |
| **Outcome** | scoping/ systematic review of all available evidence |  |

### Sources of Literature:

The following clinical databases will be searched:

1. **Medline:** https://www.ncbi.nlm.nih.gov/pubmed/
2. **Cochrane Reviews** https://www.cochranelibrary.com
3. **Embase**

Databases will be searched using Medical Search Headings (MeSH) where possible:

**Medline:**

1 exp Endoscopy, Gastrointestinal/ 98063

2 exp Endoscopy, Digestive System/ 121572

3 exp Colonoscopy/ 33796

4 exp Sigmoidoscopy/ 4879

5 exp Duodenoscopy/ 3145

6 exp Esophagoscopy/ 15352

7 exp Gastroscopy/ 17720

8 exp Proctoscopy/ 3055

9 exp Capsule Endoscopy/ 3442

10 ((GI or gastrointestinal) adj3 endoscop*).mp. 30970

11 (intestin* adj3 endoscop*).mp. 1060

12 colonoscop*.mp. 46670

13 duodenoscop*.mp. 4614

14 eosophagogastroduodenoscop*.mp. 1

15 esophagoduodenoscop*.mp. 45

16 esophagogastroduodenoscop*.mp. 4774

17 esophagoscop*.mp. 16554

18 gastroscop*.mp. 22661

19 oesophagoduodenoscop*.mp. 12

20 oesophagogastroduodenoscop*.mp. 463

21 oesophagoscop*.mp. 531

22 rectoscop*.mp.584

23 sigmoidoscop*.mp. 7485

24 (upper adj3 endoscop*).mp. 12717

25 exp Gastrointestinal Tract/ or exp Gastrointestinal Diseases/ or exp Digestive System/ 2062767

26 exp Endoscopy/392522

27 25 and 26 145466

28 gaze analys*.mp. 47

29 (eye adj2 track*).mp. 8614

30 eyetrack*.mp. 410

31 eye movement measurement*.mp. 1994

32 eye movement track*.mp. 71

33 optical track*.mp. 991

34 gaze track*.mp.284

35 Gaze control.mp. 363

36 visual track*.mp. 932

37 visual pattern*.mp. 1401

38 exp Eye Movement Measurements/ 13450

39 exp Eye Movements/ 50386

40 eye gaze.mp. 1552

41 eye movement*.mp. 55445

42 28 or 29 or 30 or 31 or 32 or 33 or 34 or 35 or 36 or 37 or 38 or 39 or 40 or 41 83353

43 1 or 2 or 3 or 4 or 5 or 6 or 7 or 8 or 9 or 10 or 11 or 12 or 13 or 14 or 15 or 16 or 17 or 18 or 19 or 20 or 21 or 22 or 23 or 24 or 27 204097

44 42 and 43 39

**Cochrane review:**

#1 MeSH descriptor: [Endoscopy, Gastrointestinal] explode all trees 4926

#2 MeSH descriptor: [Endoscopy, Digestive System] explode all trees 5800

#3 MeSH descriptor: [Colonoscopy] explode all trees 2269

#4 MeSH descriptor: [Sigmoidoscopy] explode all trees 298

#5 MeSH descriptor: [Duodenoscopy] explode all trees 184

#6 MeSH descriptor: [Duodenoscopy] explode all trees 184

#7 MeSH descriptor: [Gastroscopy] explode all trees 872

#8 MeSH descriptor: [Proctoscopy] explode all trees 68

#9 MeSH descriptor: [Capsule Endoscopy] explode all trees 139

#10 (GI or gastrointestinal) near/2 endoscop* 5400

#11 intestin* near/2 endoscop* 239

#12 colonoscop* or duodenoscop* or eosophagoduodenoscop* or eosophagogastroduodenoscop* or eosphagoscop* or esophagoduodenoscop* or esophagogastroduodenoscop* or esophagoscop* or gastroscop* or oesophagoduodenoscop* or oesophagogastroduodenoscop* or oesophagoscop* or proctoscop* or rectoscop* or sigmoidoscop* 12132

#13 upper near/2 endoscop* 1666

#14 MeSH descriptor: [Gastrointestinal Diseases] explode all trees 37665

#15 MeSH descriptor: [Gastrointestinal Tract] explode all trees 12480

#16 #14 or #15 45348

#17 MeSH descriptor: [Endoscopy] explode all trees 19324

#18 #17 and #16 5515

#19 #18 or #13 or #12 or #10 or #9 or #8 or #7 or #6 or #5 or #4 or #3 or #2 or #1 18846

#20 "gaze analysis" or "gaze analyses" 3

#21 eye near/2 track* 866

#22 eyetrack* 381

#23 "eye movement measurement" or "eye movement measurements" 63

#24 "eye movement track" or "eye movement tracking" 4

#25 "optical track" or "optical tracking" 32

#26 "gaze track" or "gaze tracking" 12

#27 "gaze control" 21

#28 "visual track" or "visual tracking" 67

#29 "visual pattern" or "visual patterns" 70

#30 MeSH descriptor: [Eye Movement Measurements] explode all trees 354

#31 MeSH descriptor: [Eye Movements] explode all trees 1344

#32 "eye gaze" 131

#33 eye movement* 4636

#34 #20 or #21 or #22 or #23 or #24 or #25 or #26 or #27 or #28 or #29 or #30 or #31 or #32 or #33 5849

#35 #19 and #34 13

**Embase:**

1 exp gastrointestinal endoscopy/172270

2 exp digestive tract endoscopy/ 245359

3 exp colonoscopy/ 95020

4 exp sigmoidoscopy/ 13208

5 exp duodenoscopy/ 4441

6 exp esophagoscopy/ 12939

7 exp gastroscopy/ 24574

8 exp capsule endoscopy/10395

9 exp esophagogastroduodenoscopy/ 18022

10 (gastrointestinal adj3 endoscop*).mp. 48406

11 (GI adj3 endoscop*).mp. 6003

12 colonoscop*.mp. 105324

13 duodenoscop*.mp. 7471

14 eosophagogastroduodenoscop*.mp. 3

15 eosphagoscop*.mp. 1

16 esophagoduodenoscop*.mp. 198

17 esophagogastroduodenoscop*.mp. 19776

18 esophagoscop*.mp. 14161

19 gastroscop*.mp. 31014

20 oesophagoduodenoscop*.mp. 23

21 oesophagogastroduodenoscop*.mp. 745

22 oesophagoscop*.mp. 611

23 proctoscop*.mp. 1239

24 rectoscop*.mp.3110

25 sigmoidoscop*.mp. 14934

26 (upper adj3 endoscop*).mp. 25551

27 exp gastrointestinal tract/ 70272

28 exp gastrointestinal disease/ 95656

29 exp endoscopy/720874

30 27 or 28 163299

31 29 and 30 22579

32 exp intestine endoscopy/ 119959

33 1 or 2 or 3 or 4 or 5 or 6 or 7 or 8 or 9 or 10 or 11 or 12 or 13 or 14 or 15 or 16 or 17 or 18 or 19 or 20 or 21 or 22 or 23 or 24 or 25 or 26 or 31 or 32 286014

34 gaze analys*.mp. 58

35 (eye adj2 track*).mp. 16668

36 exp eye tracking/ 11553

37 eyetrack*.mp. 675

38 eye movement measurement*.mp. 114

39 eye movement track*.mp. 98

40 optical track*.mp. 1377

41 gaze track*.mp.352

42 gaze control.mp. 445

43 visual track*.mp. 1164

44 visual pattern*.mp. 1633

45 exp oculography/ 18207

46 exp eye movement/ 58826

47 eye gaze.mp. 1820

48 eye movement*.mp. 76411

49 exp eye-tracking technology/ or exp eye tracking system/ 989

50 34 or 35 or 36 or 37 or 38 or 39 or 40 or 41 or 42 or 43 or 44 or 45 or 46 or 47 or 48 or 49 102889

1. 3 and 50 119

### Inclusion / Exclusion criteria

Retrieved literature will be selected according to the inclusion / exclusion criteria given below:

***Supplementary Table 2: Inclusion/ Exclusion Criteria***

| **Code** | **Inclusion criteria** |
| --- | --- |
| I1 | Studies on human subjects |
| I2 | Relevant to question |
| I3 | Peer reviewed publications or clinical trials/investigations |
| I4 | Literature published in the English language |
| **Code** | **Exclusion criteria** |
| E1 | Studies not in human subjects |
| E2 | Not relevant to question |
| E3 | Literature published in a language other than English |
| E4 | Duplicate publications |

Search summary:

***Supplementary Figure 1: Summary of studies retrieved in the Literature Search***


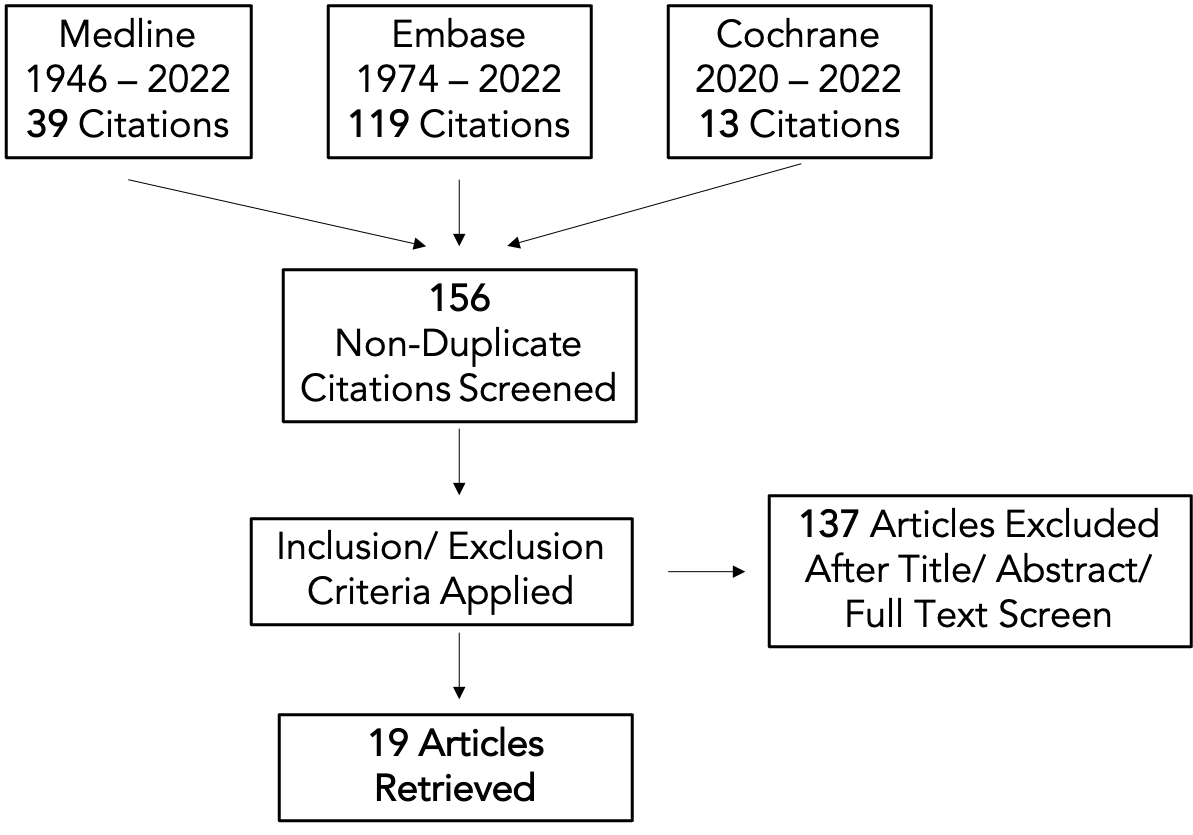

Supplement: Supplementary file 1 — Appendix S1 Eye gaze literature review: search strategy. [file DEN-35-314-s001.docx]
